# CANsec: A Practical in-Vehicle Controller Area Network Security Evaluation Tool

**DOI:** 10.3390/s20174900

**Published:** 2020-08-30

**Authors:** Haichun Zhang, Xu Meng, Xiong Zhang, Zhenglin Liu

**Affiliations:** School of Optical and Electronic Information, Huazhong University of Science and Technology, Wuhan 430074, China; d201880640@hust.edu.cn (H.Z.); M201872343@hust.edu.cn (X.M.); M201871254@hust.edu.cn (X.Z.)

**Keywords:** Internet of Vehicles (IoV), Controller Area Network (CAN), security evaluation tool

## Abstract

The Internet of Things (IoT) is an industry-recognized next intelligent life solution that increases the level of comfort, efficiency, and automation for citizens through numerous sensors, smart devices, and cloud stations connected physically. As an important application scenario of IoT, the Internet of Vehicles (IoV) plays an extremely critical role in the intelligent transportation field. In fact, the In-Vehicle Network of smart vehicles that are recognized as the core roles in intelligent transportation is currently the Controller Area Network (CAN). However, the In-Vehicle CAN bus protocol has several vulnerabilities without any encryption, authentication, or integrity checking, which severely threatens the safety of drivers and passengers. Once malicious attackers hack the vehicular gateway and obtain the access right of the CAN, they may control the vehicle based on the vulnerabilities of the CAN bus protocol. Given the severe security risk of CAN, we proposed the CANsec, a practical In-Vehicle CAN security evaluation tool that simulates malicious attacks according to major attack models to evaluate the security risk of the In-Vehicle CAN. We also show a usage case of the CANsec without knowing any information from the vehicle manufacturer.

## 1. Introduction

The development of sensors and communication technology promotes the evolution of the Internet of Things (IoT). The number of devices connected to the Internet increases rapidly around us, which constructs a network called IoT. The IoT consists of sensors, smart devices, vehicles, cloud stations, and so on. These devices are connected through various communication protocols physically and exchange data in the network, which increases the level of comfort, efficiency, and automation for citizens [1]. With the help of sensors and smart terminals, intelligent transportation, an important component of the smart city, has become an important application of IoV. As an extension of IoT, the IoV efficiently connects smart vehicles, road infrastructures, mobile devices, and the Internet, laying a foundation for building intelligent transportation.

Playing a critical role in IoV, the smart vehicle has been not only a transportation tool, but also an increasingly sophisticated computer on wheels over the past two decades, with the rapid development of automotive electronics. Current vehicles are equipped with WiFi access points, Bluetooth modules, cellular communication modules, gateways, telematics, and dozens of Electrical Control Units (ECUs) [2]. A modern vehicle, even if not fully featured, already has 70 to 100 ECUs, with over 2500 signals to transmit internally [3]. To coordinate communication among ECUs, In-Vehicle Networks (IVNs) are composed of several kinds of bus protocols. The sensors, actuators, and processors connected with several buses of IVNs provide modern information services in various scenarios according to the need of drivers and manufacturers.

**Figure 1:**
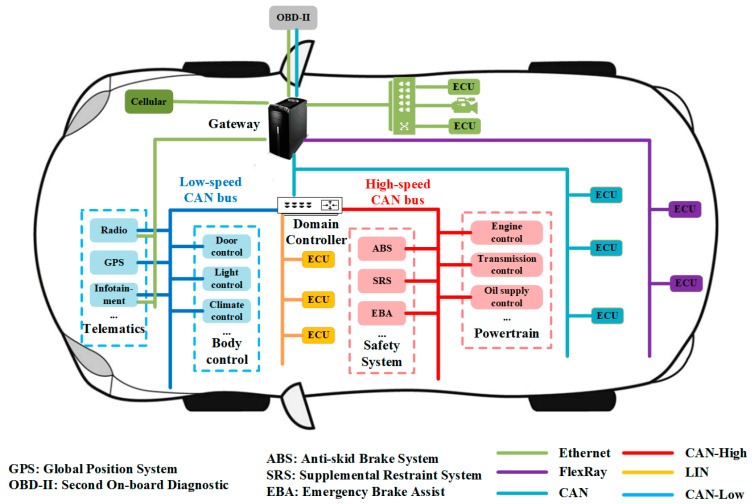
Architecture of the IVNs.

There are five bus protocols for IVN communication as demonstrated in Figure 1.

The Media Oriented System Transport (MOST) is deployed in high-end vehicles for entertainment information data transmission [4]. The Vehicle-mounted Ethernet has received a great deal of attention recently and is used in modern cars for the high-speed transmission of large amounts of data with high bandwidth and very limited latency and jitter [5]. The Local Interconnection Network (LIN) is used in low-speed data transmission scenarios without strict requirements for communication latency [4]. The FlexRay is used as the backbone of the new generation of IVN with the characteristics of efficient network utilization and system flexibility [6]. The Controller Area Network (CAN) is the de facto standard in most IVNs due to its dramatically decreased communication lines and higher data transmission reliability [7]. Through Ethernet, FlexRay, and the LIN bus protocol are also used, the CAN bus offers advantages such as cost-effective wiring, immunity to electrical interference, self-diagnosing, and error correction based on protocol characteristics, which makes the CAN bus the most common in-vehicle communication protocol [8].

However, the CAN bus was primarily designed for reliable communication without considering cybersecurity. The lack of encryption, authentication, and integrity checking introduces vulnerabilities for the CAN protocol making IVNs vulnerable to cyber-attacks, which behooves researchers to evaluate the security of the CAN bus. However, before the release of vehicular security guidelines and evaluation standards from a working group in WP.29, most of the research mainly focused on CAN bus threat analysis and security analysis methodology. Few researchers have proposed practical security assessment tools for the CAN bus. Practical evaluations of the security of the CAN must be resolved. Koscher et al. developed a CAN network analysis tool called CarShark [7]. The tool only distinguishes the critical control messages by visualization and does not evaluate the security of CAN. Huang at al. designed and developed an Attack Traffic Generation (ATG) tool for security testing of the in-vehicle CAN bus [9]. However, the evaluation items are not comprehensive for attack models. Park at al. proposed a security evaluation methodology and tool that can analyze the security level of the In-vehicle network covering only four evaluation vectors [10]. The tools in [11,12,13] only inject malicious attack messages into the CAN bus as data generators and transceivers.

The existing tools have limitations, as described above. In this paper, we designed an evaluation tool called CANsec based on a more comprehensive evaluation methodology. The evaluation methodology proposes four basic attack vectors against the CAN. In our evaluation methodology, any attack models can be constructed with four basic attack vectors. CANsec consists of 11 evaluation vectors based on target assets and basic attack vectors. The major contributions in this paper are as follows.

We define six vulnerabilities after analyzing the security characteristics of CAN. Further, based on vulnerabilities, we propose four basic attack vectors against CAN.

We propose the evaluation methodology: analyzing the target assets of CAN and constructing the evaluation vector according to four basic attack vectors and assets.

We describe the procedure of the proposed evaluation tool and provide a usage case based on a Ford car without knowing any information.

Accordingly, the rest of the paper is organized as follows. Section 2 provides a background study on the CAN, followed by Section 3, which presents a detailed vulnerability assessment of the CAN. Section 4 provides an in-depth description of the proposed evaluation tool. Section 5 discusses the results of the experiment. We reach the conclusion in Section 6.

## 2. In-Vehicle CAN Bus Protocol

The CAN is an ISO bus standard proposed in 1993 and has been the de facto standard for connecting ECUs in vehicles over the past 20 years. All ECUs are connected as nodes through a physically conventional two-wire bus, which transmits differential wired-AND signals. In this section, we focus on the CAN protocol before assessing the vulnerabilities.

**Figure 2:**
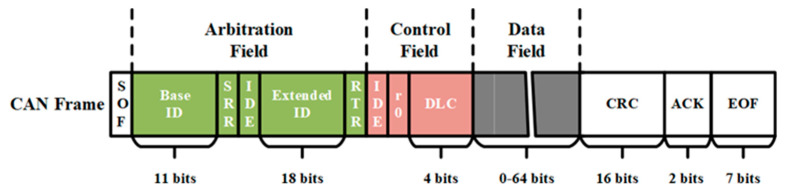
CAN frame format.

In the CAN protocol, a sender ID-based broadcast protocol [14], the CAN frames used for ECU communication consist of 7 main fields, as shown in Figure 2.

The Start of Frame (SOF) that has 1 bit informs the start of transmission. The arbitration field starts with the 11-bit ID, followed by the 1-bit Remote Transmission Request (RTR) in a standard frame or by the 1-bit Substitute Remote Request (SRR) in an extended frame. The RTR is used to distinguish the data frames (0 as dominant) from the remote request frames (1 as recessive). The SRR remains recessive to guarantee the deterministic resolution of the arbitration field between a standard frame and an extended frame. The Identifier (ID) in the arbitration field instead of an explicit address was used to identify the receivers. According to the length of ID, there are two types of CAN frames in the CAN protocol. Standard frames with an 11-bit ID and extended frames with a 29-bit ID can be simultaneously transmitted on the CAN bus.

The Control field consisting of 6 bits plays a role in displaying the properties of a data frame [15]. The 1-bit Identifier Extension (IDE) is dominant in a standard frame and recessive in an extended frame, which ensures the deterministic resolution of the contention when the first 11-bit IDs of two frames are the same. The following 4-bit Data Length Content (DLC) defines the length of the data field in bytes (0 to 64 bytes). The Cyclic Redundancy Code (CRC) can identify whether the data frame was transmitted to the receiver normally. The ACK consists of 2 bits: the first is used to record an acknowledgement from the receiver, and the other is a delimiter. The receivers report that the received frame is valid by overwriting the ACK slot with a dominant bit.

## 3. Vulnerabilities and Attack Vectors

In this sector, we analyze the intrinsic vulnerabilities of the CAN protocol and attack vectors that can be exploited to attack the In-Vehicle system. The basic attack vectors that build any attack models against the CAN in IVNs may exploit multiple CAN bus vulnerabilities. As described in Table 1, we present the mapping between vulnerabilities and attack vectors that show the vulnerabilities exploited by the specific basic attack vector.

### 3.1. Vulnerabilities

The CAN bus lacks the fundamental security mechanism in the protocol [15], which makes the vehicles vulnerable to malicious adversaries. According to CIA (Confidentiality, Integrity, Availability) security model, there are six vulnerabilities. The vulnerabilities regarding the traffic of the CAN bus include no encryption, no authentication, and no integrity checking. Moreover, the vulnerabilities introduced by the protocol characteristics of the CAN bus consist of broadcast transmission, priority-based arbitration, and limited bandwidth. No encryption violates the confidentiality principle. No integrity checking and no authentication violate the integrity principle. Priority-based Arbitration, Limited Bandwidth, and Payload make the DoS attack practical for malicious attackers, which violates the availability. The Broadcast Transmission lays the foundation for the CAN frame eavesdropping within a segment, which helps to reveal the content of the CAN frames.

(1) No Encryption. No encryption of the content in the CAN frame allows the adversaries to easily analyze the functions of the target ECU based on the historically recorded CAN frames.

(2) No Authentication. As shown in Figure 2, the CAN frame has no authentication field to indicate its source, which means a transmitter can indistinguishably transmit a CAN frame to any ECUs connected on the CAN bus. The adversaries can command a compromised ECU to take control of the target ECUs by transmitting fabricated CAN frames containing appropriate contents on the CAN bus.

(3) No Integrity Checking. The CAN frame receiver does not check the integrity of the data. The information received may be exactly different from what the sender has sent in the channel with a malicious alternation of adversaries.

(4) Broadcast Transmission. The CAN frames are both physically and logically broadcasted to all the connected ECUs. Every ECU receives the frames transmitted on the CAN bus and takes actions according to the frame ID [16]. Although manufacturers segment the CAN networks with the help of the CAN firewall, malicious ECUs can easily leverage the broadcast nature of the CAN bus to eavesdrop on the CAN frames transmitted by other ECUs within one segment.

(5) Priority-based Arbitration. The ID field of a CAN frame determines its priority. The priority-based arbitration mechanism allows a CAN frame with a smaller ID (higher priority) to be transmitted on the CAN bus while forcing all the other CAN frames to back off. If a malicious ECU asserts a dominant state on the CAN bus indefinitely, none of the legitimate ECUs can transmit any CAN frames. In this way, the adversaries can easily launch the Denial of Service (DoS) attack against IVNs.

(6) Limited Bandwidth and Payload. The high-speed CAN bus has a data rate of about 500 Kbit/s, and the payload of a CAN frame is up to 64 bits [15]. Limited by the bandwidth and the payload, the CAN bus cannot provide strong access control. For example, in order to protect ECUs against certain operations without authorization, ECUs in diagnostic services are supposed to use fixed challenges (seeds) and store the corresponding responses (keys) for the challenge-response pairs [7]. Since the length of the challenges and the responses are too short, the adversaries can crack the key of an ECU within eight days through a brute-force attack [7].

### 3.2. Attack Vectors

The CAN bus protocol has no encryption, no authentication, and no integrity checking. Furthermore, the CAN bus cannot determine whether the data was replayed by a malicious node even if a corresponding cryptographic mechanism is adopted to tackle the previous vulnerabilities. According to the above security vulnerabilities, we propose four basic attack vectors. The eavesdrop attack exploits the vulnerability of no encryption. The impersonation attack exploits the vulnerability of no authentication. The impersonation attack can manipulate the CAN frames because there is no integrity checking. The replay attack may be effective if no countermeasure has been deployed. The attackers can launch the basic attack vectors with the weakly and fully compromised ECUs. Since a practical attack model is a combination of one or more basic attack vectors, we explain how to use the weak attacker and the strong attacker to launch the attack vectors in this section.

**Figure 3:**
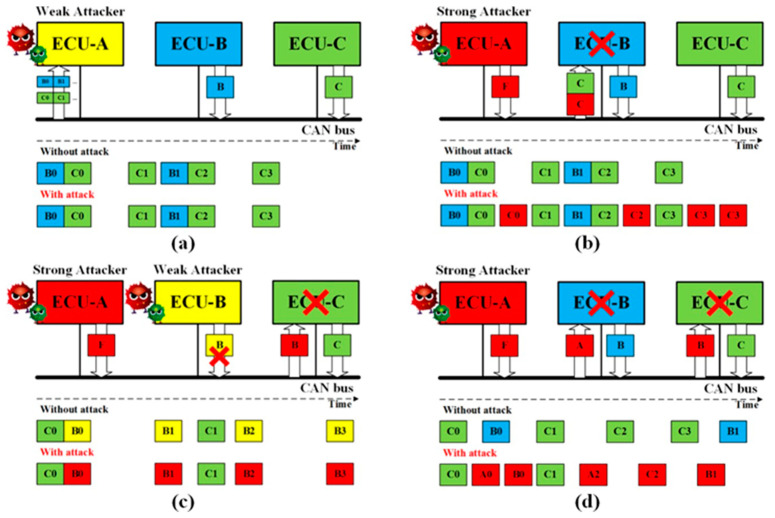
Basic four attack vectors [17]: (**a**) Eavesdrop attack; (**b**) Replay attack; (**c**) Impersonation attack; (**d**) Injection attack.

(1) Eavesdrop Attack. As mentioned before, the CAN frames are broadcasted to all ECUs without encryption. A weak attacker, ECU-A in Figure 3a, is able to eavesdrop on the CAN bus to collect and analyze the CAN frames.

Through the fuzzing test [7] on the historically recorded CAN frames, the functions of the target ECUs can be determined. Therefore, the eavesdropping attack is the foundation of all the practical attacks.

(2) Replay Attack. Without authentication and integrity for the CAN frames, a strong attack is able to launch the replay attack [18,19]. As shown in Figure 3b, a fully compromised ECU-A transmits the CAN frames received from ECU-C without modifying it. As a result, the receiver ECU-B will function abnormally under the replayed control information.

(3) Impersonation Attack. Having known the content and frequency of the CAN frames from ECU-B, the strong attack is able to launch the impersonation attack, as shown in Figure 3c [20]. The weak attacker first suspends the transmission of ECU-B, and the strong attacker then controls ECU-A to transmit CAN frames using ECU-B’s ID to manipulate the target ECU-C.

(4) Injection Attack. As shown in Figure 3d, a strong attacker ECU-A is able to inject CAN frames with arbitrary IDs and content [11]. On the one hand, the injected frames with the highest priority ID will always occupy the CAN bus [16]. On the other hand, an appropriate ID makes the target ECUs accept the content in the fabricated CAN frames.

## 4. Proposed Security Evaluation Tool

### 4.1. Evaluation Methodology

The evaluation methodology includes evaluation assets and evaluation vectors. The In-Vehicle CAN is composed of several ECUs communicating through CAN packets [21]. From the angle of the application layer, there are two main types of CAN packets. Normal packets are transmitted on the CAN bus at any given time to be interpreted as commands for receivers. Diagnostic packets are sent form diagnostic tools to communicate with ECUs only when the automotive need to be diagnosed. The ECUs and two types of CAN packets will be the targets of malicious attackers if there are no security mechanisms in the In-Vehicle CAN. There are seven major assets in the automotive CAN network: the CAN architecture, CAN frames, ECU diagnostic services, the ECU communication matrix, ECU access rights, ECU data, and ECU functions.

CANsec supports 11 evaluation vectors predefined. The evaluation vector constructed based on four basic attack vectors simulates the actual attack models against the target assets. Adversaries may launch the attack described in the evaluation vectors. Accordingly, the security evaluator can attack the target with the evaluation vector to evaluate its security. The role of each evaluation vector with the target asset is shown below.

(1) The CAN Architecture Scan. The In-Vehicle CAN network is composed of multiple sub-networks. The ECU is connected to different sub-networks depending on its function. By continuously eavesdropping on CAN data frames, the malicious attacker can infer the CAN architecture of the target vehicle according to the CAN IDs of the CAN data frames. The CAN architecture scan regards the CAN architecture as the target asset. Through the CAN architecture, the malicious attackers can obtain the location of critical ECUs in the automotive CAN networks. 

(2) ECU Drop-Off. The CAN bus has an arbitration mechanism based on ID priority. When messages of high priority are sent to the CAN bus continuously, messages sent by other senders will be blocked, resulting in a denial of service or an interruption of service for ECUs on the bus, which is called the ECU drop-off. The evaluation vector of the ECU drop-off targets the ECU function. Adversaries may take actions to deplete the CAN bus communication resources to force the ECU to fail to provide normal service.

(3) Normal packets Reverse based on the frame frequency. Reversing the normal packets reveals the communication matrix preserved by the manufacturers. With the help of the communication matrix, the adversaries can compromise the CAN bus system to control the action of the vehicles. The ECUs broadcast normal packets at a certain frequency. However, when the vehicular status changes frequently, the ECU will broadcast the corresponding normal packets at a higher frequency, which can be used to figure out the mapping between the CAN ID and the vehicular action.

(4) Normal packets Reverse based on the data bit feature. CANsec introduces eigenvalues as the length of the valid data bits in the CAN frame. For each automobile action, the evaluation tool determines the eigenvalue through a large number of statistical analyses of the corresponding CAN frames. Based on the eigenvalues, CANsec gradually changes the value of the valid data bits and analyzes the corresponding automobile actions to obtain the communication matrix.

(5) Normal packets Replay. The replay attack is an active attack based on the eavesdropping attack, and the evaluation vector can be launched on both normal packets and diagnostic packets. By eavesdropping on and recording all messages on the CAN bus when the vehicular status changes, CANsec can identify and replay the recorded messages to control the behavior of the vehicle. The replay attack regards the CAN frames as the target assets.

(6) Normal packets Fuzzing. In the fuzzy test module of CANsec, the fuzzy data generator uses the mutation mechanism to generate massive normal packets. Based on the legal normal packets collected from the vehicle, the fuzzy frames for the tests are generated by a combination of random ID and data. By monitoring a change in vehicular status while transmitting the fuzzy frames to the In-vehicle CAN, the tool can discover unknown vulnerabilities and effectively evaluate the security of the In-vehicle CAN.

(7) Diagnostic Service Scan. The Unified Diagnostic Services (UDS) defines a diagnostic packet that includes diagnostic IDs, primary services, and subfunctions. The diagnostic service scan is helpful to understand that whether the target vehicle ECU supports the specific diagnostic service, which lays an important foundation for reversing diagnosis instruction. The asset of the evaluation vector is the diagnostic service that the target vehicle provides.

(8) Diagnostic packets Reverse. The diagnostic service provided by the vehicle can be obtained through the diagnostic service scan. Further analysis of the diagnostic service parameters can reverse effective diagnostic control instructions. Malicious attackers can obtain ECU data through the diagnostic packets reverse.

(9) ECU Access. The UDS diagnostic protocol specifies that some important diagnostic services involving ECU reading and writing require the identification of external diagnostic tools. The authentication mechanism is a seed-key algorithm. The diagnostic client sends a seed request to the target vehicle and then receives a randomly generated seed. Both the diagnostic client and the target vehicle calculate the key based on the encryption algorithm defined by the manufacturer and the seed. If the client provides the correct key for the target vehicle, it will be authenticated. External clients that have passed the security authentication will access the data in the ECUs.

(10) Diagnostic packets Replay. The diagnostic packets replay is familiar with the normal packets replay. By eavesdropping on and recording all diagnostic messages on the CAN bus from the session between diagnostic tool and ECUs, CANsec can identify and replay the recorded messages to manipulate the behavior of the ECU to access the ECU or obtain the ECU data.

(11) Diagnostic packets fuzzing. The diagnostic package only comes from the session between the diagnostic tool and the ECU. It is not possible to collect a large number of diagnostic packages from the vehicle itself for mutation. Therefore, based on UDS protocol specification, CANsec generates a large amount of fuzzy diagnostic frames, which greatly improves the efficiency of the fuzzy test.

The evaluation vector builds an attack model for the target assets and simulates the actual attack scenarios. Table 2 presents the mapping between Evaluation vectors and attack vectors that show the attack vectors exploited by a specific evaluation vector.

### 4.2. In-depth Knowledge of CANsec

#### 4.2.1. Overview of CANsec

**Figure 4:**
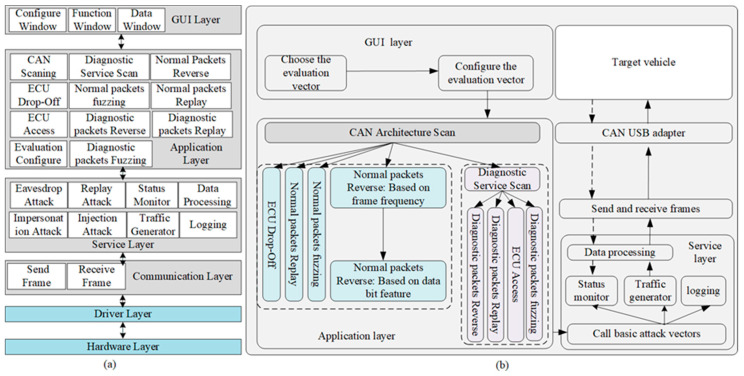
CANsec architecture: (**a**) The framework of CANsec; (**b**) Automatic evaluation flow.

As shown in Figure 4a, CANsec is composed of hardware and software written mainly in Python.

Like other tools, the tool has conventional functionality, such as traffic tracking, transmitting, logging, and monitoring. The tool supports 11 evaluation vectors that target various assets of IVNs, which comprehensively evaluates the security of CAN bus in IVNs. Users can choose the evaluation vector in the function window to execute the specific evaluation. The user-specified evaluation vector calls several of the four basic attack vectors for a combined attack to evaluate the security of the CAN. All evaluation vectors support a flexible configuration defined by the configuration window to adapt various evaluation scenarios before evaluations.

The data processing module processes the CAN traffic for display. The status monitor keeps an eye on whether the status of the target vehicle has changed when CANsec executes the fuzzy evaluation. The logging module logs critical events such as vehicular status changes and software crashes. The communication layer sends and receives the CAN application data, which is called by the upper layer for communication with the target vehicle. The hardware with the driver software, called the CAN transceiver, automatically completes the analysis and encapsulation of CAN frames, which makes the application layer focused on the CAN ID and data field. The primarily supported hardware is a cost-effective CAN USB adapter.

#### 4.2.2. Details of CANsec

As shown in Figure 4b, before the evaluation, users should choose the evaluation vector and configure the chosen evaluation vector. The CAN architecture scan must be the first evaluation item to obtain the architecture of the in-vehicle CAN. When launching the normal packets reverse item, CANsec reverses the CAN ID based on the frame frequency before reversing the communication matrix based on the bit feature. When evaluating the security of the diagnostic functionality, the tool will scan the CAN to find the diagnostic services provided by the target vehicle, which is the cornerstone of the follow-up evaluation. The user-specified evaluation vector invokes the basic attack vectors to generate test data streams to attack the target vehicle. At the same time, the tool will monitor the change of the target vehicle under test and record the evaluation log. The detailed procedure of the evaluation vector is as follows.

(1) CAN Architecture Scan. The purpose of the evaluation vector is obtaining the architecture of the In-Vehicle CAN. Users initiate the vehicle and connect the evaluation hardware device to the OBD-II port of the target vehicle. Users then eavesdrop on the CAN bus frames and record the CAN IDs that helps to indicate the architecture of the In-Vehicle CAN.
(2) ECU Drop-Off. The evaluation vector tries to deny the services of the ECU. After initiating the vehicle and connecting the evaluation hardware device to the OBD-II port of the target vehicle, users eavesdrop on the CAN bus frames and record the CAN IDs. Users send the CAN frames based on the target CAN ID at a certain frequency and record the CAN IDs again. If the CAN IDs in two records change, the attack is valid, and the CAN communication is not secure.
(3) Normal packets Reverse based on the frame frequency. The asset of the evaluation vector is the mapping between CAN IDs and vehicular actions. After initiating the vehicle and connecting the evaluation hardware device to the OBD-II port of the target vehicle, users eavesdrop on and record the CAN bus frames. After manipulating the target vehicle to trigger the vehicular action, users eavesdrop on the CAN bus communication and record the CAN frames again. By comparing the two records, users can find the CAN ID that occurs more frequently and replay the CAN frame related to the CAN ID. If the vehicular action occurs again, the CAN ID is related to the vehicular action.
(4) Normal packets Reverse based on the data bit feature. The asset of the evaluation vector is the communication matrix. After initiating the vehicle and connecting the evaluation hardware device to the OBD-II port of the target vehicle, users eavesdrop on and record the CAN bus frames. After manipulating the target vehicle to trigger the vehicular action, users eavesdrop on the CAN bus communication and record the CAN frames again. By analyzing the two records, users can figure out the communication matrix. Users then send the CAN frame constructed according to the communication matrix to the vehicle to verify if the communication matrix is correct. If the vehicular status changes as expected, the communication matrix is correct.
(5) Normal packets Replay. The purpose of the evaluation vector is verifying if the replay attack against the In-Vehicle is valid. After initiating the vehicle and connecting the evaluation hardware device to the OBD-II port of the target vehicle, users eavesdrop on and record the CAN bus frames. Users replay the CAN frame recorded to the In-Vehicle and observe the vehicular action. If the vehicle acts as the CAN frame defined, the replay attack is valid.
(6) Normal packets fuzzing. The evaluation vector tries to find unknown vulnerabilities of the In-Vehicle. After initiating the vehicle and connecting the evaluation hardware device to the OBD-II port of the target vehicle, users eavesdrop on and record the CAN bus frames. Users then construct massive fuzzy normal packets based on the mutation of recorded packets, send the fuzzy normal packets, and observe the status of the vehicle. If the vehicle crashes and has another abnormal status, the fuzzy test is valid.
(7) Diagnostic Service Scan. The evaluation vector aims to figure out the diagnostic services of the ECUs. After initiating the vehicle and connecting the evaluation hardware device to the OBD-II port of the target vehicle, users construct the diagnostic request according to the UDS protocol. The request should cover all diagnostic services by configuring the diagnostic frames. Users send the diagnostic frames constructed to the vehicle and record the response from the vehicle and determine if the vehicle opens the corresponding diagnostic service according to the response code from the vehicle and the UDS protocol specification.
(8) Diagnostic packets Reverse. The purpose of the evaluation vector is obtaining the diagnostic command. After initiating the vehicle and connecting the evaluation hardware device to the OBD-II port of the target vehicle, users construct the diagnostic packets according to the UDS protocol by configuring the data field of the diagnostic CAN frame, send the diagnostic packets to the vehicle, and verify whether the vehicle responds with the diagnostic CAN frame correctly. If the vehicle responds with the diagnostic CAN frame correctly, we reverse the diagnostic command correctly.
(9) ECU Access. The asset of the evaluation vector is the access right of the ECU. After initiating the vehicle and connecting the evaluation hardware device to the OBD-II port of the target vehicle, users choose the diagnostic session mode that needs authentication, send the authentication request constructed based on the UDS protocol to the vehicle, and record the seed from the vehicle. Users then calculate the key based on the seed and send the key to the vehicle. If the CANsec can access the ECU in security mode, the attack is valid.
(10) Diagnostic packets Replay. The evaluation vector tries to verify whether the diagnostic replay attack for the In-Vehicle is valid. After initiating the vehicle and connecting the evaluation hardware device to the OBD-II port of the target vehicle, CANsec eavesdrops on and records the session between the diagnostic tool and vehicle. Users then replay the diagnostic packets recorded to the In-Vehicle CAN and observe the vehicular action. If the vehicle response to the diagnostic packets, the replay attack is valid.
(11) Diagnostic packets fuzzing. The evaluation vector tries to find unknown vulnerabilities in the diagnostic services. After initiating the vehicle and connecting the evaluation hardware device to the OBD-II port of the target vehicle, CANsec constructs massive fuzzy normal packets based on the UDS protocol, sends fuzzy normal packets, and observes the status of the vehicle. If the vehicle crashes and has another abnormal status, the fuzzy test is valid.

#### 4.2.3. Advantages of CANsec

According to Table 3, most tools, like CarShark, are only CAN traffic generation tools. ATG and the tool in [10] are familiar with CANsec. However, the ATG and the tool in [10] can only launch a basic attack, such as an injection attack, a replay attack, or a DoS attack. They do not construct more elaborate attack scenarios based on basic attack vectors. CANsec is not only an attack traffic generation tool, but also a security evaluation tool that includes 11 evaluation vectors. Reversing the CAN traffic is a key feature of CANsec that other tools do not have. With the help of reverse traffic functionality, CANsec can obtain the communication matrix to manipulate the CAN traffic more precisely. In particular, CANsec has the following advantages:

(1) CANsec allows users to configure the evaluation flexibly after selecting the evaluation vectors.
(2) CANsec supports the 11 evaluation vectors defined above based on four basic attack vectors. And CANsec provides a comprehensive assessment of IVNs, including normal packets assessment and diagnostic packets assessment.
(3) CANsec can monitor the change in vehicular status and log the evaluation activity.
(4) Besides the replay attack, the DoS attack, and the fuzzing attack, CANsec can also reverse CAN traffic to obtain the communication matrix of the manufacturers.

## 5. Experiments

To verify the function of the proposed evaluation tool, we conducted an experiment based on a Ford vehicle. We found that the vehicle consists of two types of CAN bus, a high-speed CAN bus with 500 kbit/s and a low-speed CAN bus with 250 kbit/s. The PCM, the instrument panel, the PSCM, and the ABS are connected to a high-speed CAN bus. The electronic control unit of the turn signal, the electronic control unit for the door lock, and the air conditioning electronic control unit are connected to a low-speed CAN bus.

**Figure 5:**
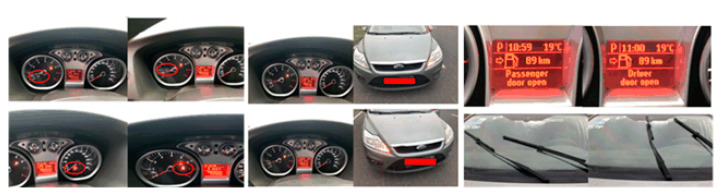
Replay attack against the Ford vehicle.

As for the evaluation of the replay attack, our experiment captured 1000 CAN frames with the help of CANsec, which consists of a CAN USB adapter and corresponding application software that can count the number of CAN frames received after triggering vehicular actions. After receiving frames, the tool replays them. The result indicated that the replay attack against the instrument panel was valid. Figure 5 shows that the engine speed on the dashboard, the turn signal on the dashboard, the door status on the dashboard, and the wiper status can reappear under a replay attack. The success of the replay attack experiment reveals the security vulnerability of the CAN bus broadcasting mechanism.

**Figure 6:**
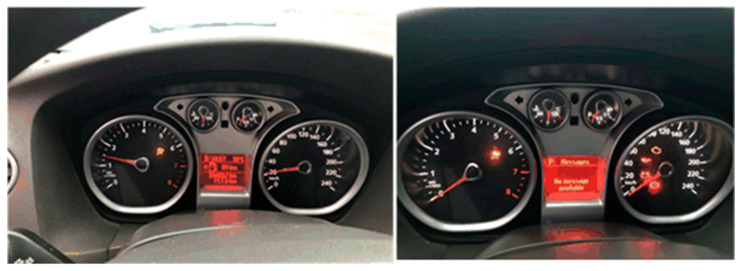
Fuzzy attack against the Ford vehicle.

In the experiment, a fuzzy attack test was carried out on the Ford vehicle, and a large number of normal and diagnostic packets were continuously broadcast to the CAN bus in the vehicle. The dashboard of the vehicle appears an abnormal display state under the attack, shown in Figure 6.

The communication matrix from the experiment is displayed in Table 4. In the Ford vehicle, the communication matrix can control the light system and the door of the vehicle physically. However, the communication matrix only controls the dashboard status of the back gear, clutch, and engine. In addition, our experiment showed that the instrument panel has a defense mechanism for handling message conflicts. However, the mechanism introduces the risk of DoS.

The diagnostic service provided in the Ford vehicle is shown in Table 5. According to the result, we can conclude that Request ID = Respond ID +0 × 08. The table lists the primary diagnostic service supported by a specific diagnostic request ID. The primary diagnostic services provide methods defined in the UDS protocol for malicious attackers to manipulate the vehicle.

## 6. Conclusions

The modern automobile is an important scene of IoT technology, which includes a large number of sensors, actuators, and processors. As the main bus to connect electronic devices, the CAN bus is the actual bus network of IVNs. However, due to the lack of corresponding security mechanisms, many security vulnerabilities have been introduced into the IVNs, which has caused serious risks to the life and property safety of members in vehicles. Although vehicle testing technology has made great progress, there is still a lack of relevant vehicle safety assessment tools in the market. Existing testing tools also have problems. In this paper, we propose a vehicle CAN network security assessment tool, CANsec, which is designed based on the assessment methodology proposed. CANsec constructs the evaluation vector according to the attack vector and target assets of IVNs. We provide a comprehensive description of the evaluation tool and its key features and evaluate the performance with a real vehicle. The tool can generate attack traffic automatically with a flexible configuration and log the critical events while conducting the security evaluation. In addition, we conducted experiments using an actual Ford vehicle without information from manufacturers to evaluate the accuracy of CANsec. As a result of the experiments, we found several vulnerabilities of the Ford vehicle through penetration test items defined in CANsec. Fortunately, vehicles equipped with gateway ECUs can prevent all attacks except replay attacks based on diagnostic tools. Through experiments, we concluded that CANsec could evaluate the security of the in-vehicle network using the proposed evaluation method to find out the vulnerabilities, which can help to promote the design of the vehicle.

## Figures and Tables

**Table 1:** Mapping between vulnerabilities and attack vectors.

Attack Vectors	No Encryption	No Authentication	No Integrity Checking	Broadcast Transmission	Priority-based Arbitration	Limited Bandwidth and Payload
Eavesdropping Attack	●	●		●		
Replay Attack		●	●		●	
Impersonation Attack	●	●		●	●	
Injection Attack	●	●	●	●	●	●

**Table 2:** Mapping between evaluation vectors and attack vectors.

**Evaluation Vectors**	**Eavesdropping Attack**	**Replay Attack**	**Impersonation Attack**	**Injection Attack**
CAN Architecture Scan	●			
ECU Drop-Off	●		●	●
Normal packets Reverse: Based on frame frequency	●	●		
Normal packets Reverse: Based on data bit feature	●	●		
Normal packets Replay	●	●		
Normal packets fuzzing	●			●
Diagnostic Service Scan	●		●	
Diagnostic packets Reverse	●		●	
ECU Access	●		●	
Diagnostic packets Replay	●	●		
Diagnostic packets fuzzing	●	●		●

**Table 3:** Comparison of different CAN bus tools.

Tool	Tracing Traffic	Reverse Traffic	Packets Generation	Configuration	Logging	Monitor	System
ATG [9]	✓		✓	✓	✓	✓	Linux, Win
CarShark [7]	✓		✓				Win
CANUtils	✓		✓		✓		Linux
BusMaster	✓		✓		✓		Win
CANoe	✓		✓	✓	✓		Win
SavvyCAN	✓		✓	✓	✓		Linux, Win
OCTAN	✓		✓	✓	✓		Win
Tool [10]	✓		✓	✓	✓	✓	Win
CANsec	✓	✓	✓	✓	✓	✓	Linux, Win

**Table 4:** Communication matrix of Ford vehicle.

CAN ID	Data Field	Functionality
0x231	Byte0 = 0xe1, Byte1 = 0x69	Turn on the back gear
0x235	Byte0 = 0x00	Open or close the door
0x265	Byte0 = 0x20	Turn on the left turn signal
0x265	Byte0 = 0x40	Turn on the right turn signal
0x285	0xff 0xff 0xff 0xff 0xff 0xff 0xff 0xff	Air-conditioning heating mode
0x240	Byte4 = 0x9a	Clutch down
0x60d	Byte1 = 0x01	Open the fog lamp
0x60d	Byte1 = 0x08	Turn on high beam
0x358	Byte0 = 0x40	Car horns
0x201	Byte0, Byte1	The vehicle speed
0x201	Byte4, Byte5	The engine speed

**Table 5:** Diagnostic services of the Ford vehicle.

Diagnostic Request ID	Diagnostic Response ID	The Primary Service ID
0x726	0x72e	0x10 0x11 0x14 0x18 0x21 0x22 0x28 0x2f 0x310x32 0x33 0x3b 0x3e 0x3f 0x85 0xb1
0x737	0x73f	0x14 0x18 0x28 0x31 0x32 0x33 0x3b 0x3e 0x3f 0x85 0xb1
0x740	0x748	0x10 0x14 0x18 0x21 0x22 0x27 0x28 0x2f 0x31 0x32 0x33 0x34 0x35 0x36 0x37 0x3b 0x3e 0x3f 0x85 0xb1
0x741	0x749	0x10 0x14 0x18 0x21 0x22 0x27 0x28 0x2f 0x31 0x32 0x33 0x34 0x35 0x36 0x37 0x3b 0x3e 0x3f 0x85 0xb1
0x742	0x74a	0x10 0x14 0x18 0x21 0x22 0x27 0x28 0x2f 0x31 0x32 0x33 0x34 0x35 0x36 0x37 0x3b 0x3e 0x3f 0x85 0xb1
0x743	0x74b	0x10 0x14 0x18 0x21 0x22 0x27 0x28 0x2f 0x31 0x32 0x33 0x34 0x35 0x36 0x37 0x3b 0x3e 0x3f 0x85 0xb1

## References

[1] Hassija V., Chamola V., Saxena V., Jain D., Goyal P., Sikdar B. (2019). A Survey on IoT Security: Application Areas, Security Threats, and Solution Architectures. IEEE Access.

[2] Kelarestaghi K.B., Foruhandeh M., Heaslip K., Gerdes R. (2019). Intelligent Transportation System Security: Impact-Oriented Risk Assessment of In-Vehicle Networks. IEEE Intell. Transp. Syst. Mag..

[3] Kimm H., Ham H. Integrated fault tolerant system for automotive bus networks. Proceedings of the 2010 Second International Conference on Computer Engineering and Applications.

[4] Huang J., Zhao M., Zhou Y., Xing C.-C. (2018). In-Vehicle Networking: Protocols, Challenges, and Solutions. IEEE Netw..

[5] Carnevale B., Fanucci L., Bisase S., Hunjan H. (2018). Macsec-based security for automotive ethernet backbones. J. Circuits Syst. Comput..

[6] Dvořák J., Hanzálek Z. (2016). Using two independent channels with gateway for FlexRay static segment scheduling. IEEE Trans. Ind. Inform..

[7] Koscher K., Czeskis A., Roesner F., Patel S., Kohno T., Checkoway S., McCoy D., Kantor B., Anderson D., Shacham H. Experimental Security Analysis of a Modern Automobile. Proceedings of the 2010 IEEE Symposium on Security and Privacy.

[8] Bozdal M., Samie M., Aslam S., Jennions I. (2020). Evaluation of CAN Bus Security Challenges. Sensors.

[9] Huang T., Zhou J., Bytes A. ATG: An attack traffic generation tool for security testing of in-vehicle CAN bus. Proceedings of the 13th International Conference on Availability, Reliability and Security.

[10] Park H.B., Kim Y., Jeon J., Moon H.S., Woo S. (2019). Practical Methodology for In-Vehicle CAN Security Evaluation. J. Internet Serv. Inf. Secur. (JISIS).

[11] Cho K.T., Shin K.G. Fingerprinting electronic control units for vehicle intrusion detection. Proceedings of the 25th USENIX Security Symposium (USENIX Security 16).

[12] Kang M.J., Kang J.W. (2016). Intrusion detection system using deep neural network for in-vehicle network security. PLoS ONE.

[13] Taylor A., Leblanc S.P., Japkowicz N. Anomaly Detection in Automobile Control Network Data with Long Short-Term Memory Networks. Proceedings of the 2016 IEEE International Conference on Data Science and Advanced Analytics (DSAA).

[14] Di Natale M., Zeng H., Giusto P., Ghosal A. (2012). Understanding and Using the Controller Area Network Communication Protocol: Theory and Practice.

[15] Avatefipour O., Malik H. (2018). State-of-the-art survey on in-vehicle network communication (CAN-Bus) security and vulnerabilities. arXiv.

[16] Liu J., Zhang S., Sun W., Shi Y. (2017). In-Vehicle Network Attacks and Countermeasures: Challenges and Future Directions. IEEE Netw..

[17] Ying X., Bernieri G., Conti M., Poovendran R. TACAN: Transmitter authentication through covert channels in controller area networks. Proceedings of the 10th ACM/IEEE International Conference on Cyber-Physical Systems.

[18] Hazem A., Fahmy H.A. Lcap-a lightweight can authentication protocol for securing in-vehicle networks. Proceedings of the 10th escar Embedded Security in Cars Conference.

[19] Noureldeen P., Azer M.A., Refaat A., Alam M. Replay attack on lightweight CAN authentication protocol. Proceedings of the 2017 12th International Conference on Computer Engineering and Systems (ICCES).

[20] Lin C.-W., Sangiovanni-Vincentelli A. Cyber-Security for the Controller Area Network (CAN) Communication Protocol. Proceedings of the 2012 International Conference on Cyber Security.

[21] Takahashi J., Tanaka M., Fuji H., Narita T., Matsumoto S., Sato H. (2019). Automotive Security on Abnormal Vehicle Behavior Using Only Fabricated Informative CAN Messages. J. Inf. Process..

